# Unloading the excess baggage: key informant interviews with Malaysian stakeholders on healthcare disinvestment initiatives

**DOI:** 10.1017/S0266462325103437

**Published:** 2026-01-05

**Authors:** Hanin Farhana Kamaruzaman, Eleanor Grieve, Evi Germeni, Habibah Kamaruzaman, Erni Zurina Romli, Ku Nurhasni Ku Abd Rahim, Izzuna Mudla Mohamed Ghazali, Sit Wai Lee, Mohammed Hirman Abdullah, Olivia Wu

**Affiliations:** 1 Health Economics and Health Technology Assessment (HEHTA), School of Health and Wellbeing, University of Glasgow, UK; 2 Malaysian Health Technology Assessment Section (MaHTAS), Medical Development Division, Ministry of Health, Malaysia; 3 Faculty of Pharmacy, University Sultan Zainal Abidin (UNISZA), Malaysia; 4 Medical Development Division, Ministry of Health, Malaysia

**Keywords:** qualitative research, disinvestment, stakeholder participation, low-value care, equity

## Abstract

**Objectives:**

This study aims to explore the perspectives on disinvestment of low-value care and interventions in Malaysia’s healthcare system, with a focus on establishing the criteria for assessing disinvestment candidates, identifying potential barriers, and proposing strategies to improve the acceptance and effective implementation of disinvestment.

**Methods:**

Between March and May 2023, we conducted online, semistructured interviews with seventeen Malaysian healthcare stakeholders with different professional roles at various levels of governance and decision making. Participants were recruited through a mix of purposive and snowballing sampling. Interviews were transcribed verbatim and analyzed using inductive thematic approach in Atlas.ti.

**Results:**

We identified four major themes: *disinvestment as a catalyst for efficient resource allocation*; *disinvestment as a justifiable way of cutting budgets*; *challenges and barriers in implementation*; and *strategies for value-based assessment and effective implementation.* Stakeholders viewed disinvestment both optimistically and skeptically in terms of its implementation but were unanimous in including equity as a key component in decision making. Practical challenges and uncertainty among healthcare professionals emerged as significant barriers to implementing disinvestment initiatives in Malaysia.

**Conclusions:**

Malaysian stakeholders viewed disinvestment as both an opportunity to improve resource allocation and a source of concern due to potential negative consequences and system readiness. This study identified strategies to support value-based assessment and implementation, underscoring the need for accountability and collaboration. Although current disinvestment efforts in Malaysia remain limited and undocumented, the thematic framework developed offers transferable insights and a structured lens for assessing readiness. These stakeholder-derived themes can guide other countries in designing transparent, equitable, and context-sensitive disinvestment processes.

## Introduction

Healthcare systems worldwide face the persistent challenge of allocating limited resources to achieve optimal health outcomes. In this context, healthcare disinvestment, defined as *“the processes of (partially or completely) withdrawing health resources from any existing health care practices, procedures, technologies or pharmaceuticals that are deemed to deliver little or no health gain for their cost, and thus are not efficient health resource allocations”* (1), has emerged as a viable strategy for improving efficiency and sustainability. However, the success of active disinvestment initiatives often hinges on the level of support they garner from relevant stakeholders, including healthcare professionals, policy makers, and the public (2).

Early engagement with stakeholders in designing disinvestment policies and implementation frameworks fosters collaboration, builds trust, aligns priorities, and enhances the likelihood of successful implementation (3;4). Existing lists of low-value care (LVC) and ongoing disinvestment programs are highlighted in our earlier scoping review (5), which identified international initiatives such as the Choosing Wisely campaign by the American Academy of Family Physicians in the United States. However, challenges remain in systematically assessing and prioritizing candidates for disinvestment within healthcare systems.

Health technology assessment (HTA) has been widely implemented globally to inform healthcare decision making. Strengthening stakeholders’ understanding of HTA can enhance healthcare capacity and promote equitable access in Asia (6). Conventionally, HTA focuses on evaluating new technologies, with limited emphasis on disinvestment. This often enables continued use of LVC, that is, tests or treatments with no proven benefit or where evidence shows more harm than benefit (4). Further, LVC displaces higher-value interventions (7). Therefore, assessing LVC and other disinvestment candidates requires broader criteria and a clear understanding of contextual enablers and barriers to ensure successful de-implementation (8).

A thorough understanding of the local context is essential in promoting the acceptability of any new policy initiatives (9). Introducing disinvestment as a policy component of value-based health care requires qualitative insights and diverse stakeholder perspectives to complement existing evidence. The present study forms part of mixed-methods research efforts exploring Malaysian healthcare stakeholders’ perceptions, practices, and acceptability of disinvestment. Previously, we conducted an online survey to gather broad insights into stakeholders’ views on disinvestment (10). To delve deeper, that research was augmented with the present study through key informant interviews (KII) to uncover detailed perspectives on priority setting and resource allocation within Malaysia’s healthcare system. Specifically, it aimed to establish criteria for assessing disinvestment candidates, identify barriers, and propose strategies to support the integration of disinvestment into Malaysia’s healthcare framework.

## Methods

### Study design, setting, and participant recruitment

This study was conducted in collaboration with the Malaysian Health Technology Assessment Section (MaHTAS), a government-based HTA agency in the Ministry of Health (MOH), to address the following questions:

1. *How do Malaysian stakeholders perceive disinvestment initiatives in the context of healthcare decision making and resource allocation?*
2. *How do healthcare professionals make decisions on stopping funding or disinvesting LVC at different levels of governance in the Malaysian healthcare system?*
3. *What are the facilitators and barriers to the implementation of disinvestment or de-implementation of LVC in Malaysia?*

The target population for the study was described in detail in our online survey using purposive sampling, which included the Malaysian healthcare stakeholders involved in resource allocation and decision making at various levels of governance (10). In total, 341 survey invitations were sent to stakeholders in Malaysia, identified through initial mapping and supplemented by contacts from Web sites, social media, and snowball referrals. Survey respondents were invited to provide contact details for follow-up interviews on healthcare disinvestment, and additional participants were recruited through snowball sampling via interviewees, professional contacts, and experts.

### Procedure

Survey respondents who agreed to participate in follow-up interviews were contacted via email to confirm their interest and arrange an interview appointment. In addition, participants recommended from snowballing were personally approached by email and provided with a brief overview of the research topic before being invited to participate in the KII. Upon agreement, the participant information sheet and consent form were emailed to them. A semistructured interview guide (Supplement 1) was developed from a literature review, research team discussions, and responses to the preceding survey. The guide was pilot-tested with two participants and refined accordingly. Informed consent was obtained from all participants prior to their interviews.

Seventeen one-on-one interviews were conducted by H.F.K. (lead researcher trained in qualitative study) via Zoom from March to May 2023, primarily in English. As semistructured interviews, discussions varied with questions rephrased and probes used as needed. Interviews lasted 30–70 minutes (average of 35 minutes). All participants consented to be digitally recorded, with audio recordings retained only for analysis in a password-protected folder.

### Data analysis

All recordings were transcribed verbatim by H.F.K., with the aid of Zoom’s transcription software, adhering to the standardized protocol as recommended by qualitative research methodology guidelines (11;12). To protect participants’ confidentiality, identifying information was replaced with pseudonyms. Twelve out of the seventeen transcripts were verified by two other trained qualitative researchers (E.Z.R. and Hb.K.) to ensure accuracy and completeness. Each transcript was returned to the respective participant for content review, and the finalized versions were uploaded into the qualitative data analysis software, Atlas.ti (version 24).

Initially, the lead researcher independently conducted open coding of transcripts to generate a preliminary coding framework. After two coding rounds, coded quotations were summarized in Excel (Microsoft Corporation) for review by the research team. The list of codes was refined collaboratively to resolve redundancies and improve clarity.

Using an inductive thematic approach, codes were grouped into themes and subthemes, which were reviewed by an experienced qualitative researcher (Ev.G.) and refined through ongoing consultation with supervisors (O.W. and El.G.) to maintain reflexivity (13) and link findings to stakeholders’ perspectives. The COnsolidated criteria for REporting Qualitative research (COREQ) checklist (11) was used for the reporting of the findings (Supplement 2).

### Ethical considerations

Ethical approval to this study was granted by the Medical Research and Ethics Committee, MOH Malaysia (registration identifier NMRR-ID-22-02570-6PR-IIR) and the Research Ethics Committee of the College of Medical, Veterinary and Life Sciences of the University of Glasgow (ethical approval identifier 200220048).

## Results

### Participant characteristics

In total, twenty-nine invitation emails were sent to the identified participants, of whom seventeen fully participated in the study. Four candidates initially expressed interest to participate but declined before the interview due to health issues, personal reasons, and demanding work schedules. Participants were categorized based on their primary role in healthcare services, namely program coordinators at MOH (PRGM), hospital directors and administrators (HOSP), clinical care providers at hospitals and health clinics (CLIN), and researchers at research institutes and hospitals (RSCH).

Table 1 outlines the characteristics of study participants. Six participants were program managers at the MOH, overseeing public health, primary care planning, hospital and clinical services, health financing, and a state health department. Others included four hospital administrators, four clinical care providers (cardiologist, family medicine specialist, pharmacist, allied health professional), and three researchers. Participants’ experience varied, with most (n = 9) having over ten years in healthcare decision making.

**Table 1.** Characteristics of interview participants (n = 17)

Participant	Experience	Roles in health care	Level of decision making
PRGM01	15 years	Program manager, health policy and strategic planning, technical advisory	National, regional
PRGM02	8 years	Program manager	National
PRGM03	>10 years	Program manager, health policy and strategic planning, technical advisory	National
PRGM04	8 years	Program manager, health policy and strategic planning, technical advisory	National
PRGM05	12 years	Program manager, health policy and strategic planning, technical advisory	National
PRGM06	13 years	Program manager	Regional, state
HOSP01	13 years	Hospital management and administration	National, regional, state, local healthcare facility
HOSP02	7 years	Hospital management and administration	State, local healthcare facility
HOSP03	4 years	Hospital management and administration	Local healthcare facility
HOSP04	7 years	Hospital management and administration	Local healthcare facility
CLIN01	14 years	Clinical care provider, healthcare research, technical input, leader of professional society	National, regional, local healthcare facility
CLIN02	8 years	Clinical care provider, technical input/advisory	Local healthcare facility
CLIN03	14 years	Clinical care provider, technical input/advisory	Regional, state, local healthcare facility
CLIN04	10 years	Clinical care provider, healthcare research	Local healthcare facility
RSCH01	6 years	Program manager, healthcare research	National, state
RSCH02	>10 years	Health policy and strategic planning, healthcare research	National, state
RSCH03	4 years	Healthcare research	Local healthcare facility

### Key findings

**Figure 1.**
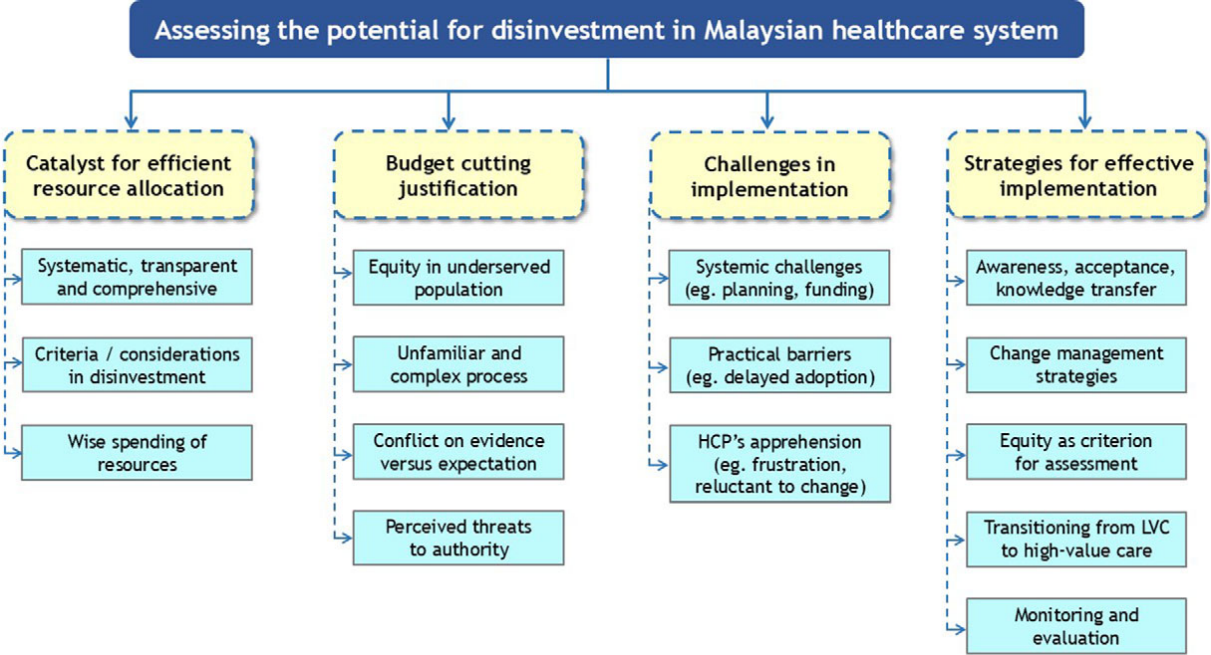
Summary of themes and subthemes from key informant interviews. *(Major themes are the yellow boxes. Subthemes [light blue boxes] that were identified most frequently are listed first within their respective groups.)*

The initial inductive thematic analysis generated 101 codes, which were later refined to 63 to better reflect the data. Four major themes identified from the KII were *disinvestment as a catalyst for efficient resource allocation; disinvestment as a justification for budget cutting*; *challenges and barriers toward disinvestment;* and *strategies for value-based assessment and effective implementation.* The major themes and subthemes are summarized in Figure 1. Although the study included seventeen interviews, the quotations presented in the following sections are not intended to be broadly representative of all stakeholder groups. Rather, they are used to illustrate the range and depth of perspectives expressed by participants and to exemplify how these views informed the development of themes and subthemes. These excerpts should therefore be interpreted as illustrative rather than exhaustive of the viewpoints within the wider population.

#### Theme 1: Disinvestment as a catalyst for efficient resource allocation

Most participants had an optimistic perspective on disinvestment, noting its potential to strengthen resource allocation when implemented holistically with stakeholder involvement. They emphasized the need for clear criteria to ensure fairness and alignment with healthcare priorities. This theme encapsulates a forward-looking view, with participants envisioning disinvestment as not just a cost-cutting measure but a strategic pathway to investing in advancements that offer higher value to both patients and providers.

##### Systematic, transparent, and comprehensive process

When examining the key elements of disinvestment, respondents concurred that evaluating existing elements in the treatment list or care provision necessitates a systematic, comprehensive, and transparent approach. Participants expressed that:

*First, the analysis itself. And then we bring that evidence to our stakeholders and get feedback from them, whether it is implementable or not. It must be systematic. (PRGM05)*

*The principle on which we need to focus and prioritize is first, transparency. For example, in the pharmaceutical’s disinvestment, we need to be transparent about the evidence, the cost-effectiveness. (CLIN03)*

Moreover, active involvement of stakeholders, particularly clinicians, enhances understanding, fosters acceptance and autonomy among healthcare practitioners, and promotes greater commitment to disinvestment decisions.

*…for engagement between the decision-maker and the ground [clinical care providers], I expect the uptake would be faster and it will increase response. Secondly, this exercise would increase knowledge and awareness. (PRGM02)*

##### Criteria and considerations in disinvestment

Participants highlighted several key criteria as they reflected on the assessment process for disinvestment. These criteria included clinical effectiveness, safety, cost and cost-effectiveness of technology, and technology’s lifecycle to determine its relevance and potential obsolescence.

*…the evidence on clinical effectiveness, which for me is the main [criterion]. Other than this and also budget, we should also consider the accessibility of the medications and also value. Does the medication give value to the clients? (CLIN03)*

*Probably we do have to look at whether the technologies are already obsolete. […] If, let’s say, you want to disinvest an old technology or product and then invest in some new technology, you probably have to look at your budget. (RSCH01)*

Participants emphasized the importance of patient preferences in considering values and willingness to accept the intervention by sharing their experience on the cervical screening program that was implemented:

*Take self-sampling using HPV-DNA, for example. If the result is negative, it is safe not to screen for at least another five years. They (the patients) prefer it this way. Although it is more expensive than doing the conventional Pap smear, it frees the patients from frequent check-up. Unlike Pap smear screening where they have to do it yearly. (PRGM04)*

Although explicit criteria are important, the source of evidence supporting disinvestment decisions is also essential. These sources of evidence include published reports of randomized clinical trials and other studies, evidence-based clinical practice guidelines, and real-world data pertaining to the use of technologies or services. In addition, expert opinion and practical experience may provide useful perspectives and prompt reassessment of technologies, including when new information emerges from conferences or scientific meetings.

*We should also look into the national guidelines, literature, expert opinions, and others before we want to discontinue or continue with it. Sometimes they [clinicians] are also responsible for reviewing clinical practice guidelines, and they attend conferences overseas. So certain things that they get from there are like, ‘This [practice] may no longer be feasible’. Therefore, they would like to stop it, and they will give us the suggestion. (HOSP01)*

##### Wise spending of resources

Participants expressed strong support for disinvestment, emphasizing its critical role in ensuring optimal use of limited resources. They assert confidence in the benefits of such initiatives, especially in promoting more efficient healthcare resource allocation. Acknowledging resource constraints, participants perceived disinvestment as an opportunity to redirect resources toward high-value technologies, replacing LVC. They regarded concerns about increased workload or administrative demands as minor compared to the substantial benefits disinvestment can bring.

*I do not see a very big resistance to no longer practice what you have done 50 years ago, because it’s no longer relevant! I don’t think anybody would be very resistant towards disinvesting it. With that money, you can redirect somewhere where it gives a higher impact. (HOSP01)*

#### Theme 2: Disinvestment as a justification for budget cutting

This theme encapsulates the apprehensions and skepticism surrounding disinvestment, frequently misconstrued as a justification for budget cuts rather than a strategic effort to enhance efficiency by reallocating funds to higher-value interventions. The complex nature of disinvestment, which requires specialized skills and expertise, may intensify resistance among healthcare providers. Some participants expressed concern that removing certain treatments could harm vulnerable or disadvantaged groups. Emotional language and reflective pauses were occasionally observed when participants described difficult or unfavorable decision-making experiences.

##### Equity issues in underserved population

Participants expressed worries regarding equity among the underserved population by giving examples of rare diseases:

*Oh, this one is very hard! Let’s just talk about rare diseases. Just the costs. That’s huge! And if you were to talk about the amount of money that we’re spending in Malaysia. We know we cannot give even the bare minimum. What we can do now is service some of the people living with rare diseases, but that’s not even all. And now, we want to take away things from them? (PRGM01)*

##### Unfamiliar and complex process

Participants viewed their unfamiliarity with disinvestment as a significant challenge for stakeholders, highlighting the absence of clear guidance as a critical concern.

*I think disinvestment is something that we are not very familiar with in our Malaysian settings. And we don’t really know the cut-off. The guidelines… hmm… I’m not sure whether there’s a national guideline for disinvestment. (CLIN04)*

In addition, limited expertise and lack of experienced personnel hinder the capacity to effectively implement and manage disinvestment decisions.

*My concern is, we are still at a very infantile stage of setting this whole thing up. One of the biggest issues that I would think is, do we have enough experts? People with, you know, experience with disinvestment to actually look and customize that to our local population. (RSCH03)*

Moreover, participants believed that a lack of experience and the complexity of decision making in disinvestment will hinder the process of gaining political will and acceptability among stakeholders:

*Do we have the political appetite to make this difficult decision? And the other thing is, do we have the skill set in our country to take the local context into consideration, when making the decision? Because the decision cannot be just purely on technical grounds. (PRGM01)*

##### Conflict on evidence versus expectation

One participant recounted a prior experience in reconciling the ethical obligations of a clinician with fulfilling patients’ expectations regarding medication. Despite robust evidence showing a lack of benefit, there is apprehension that disinvestment could lead to patient resistance, as patients have become accustomed to certain medications and may expect continued access.

*For example, the use of Neurobion in treating neuropathy. Even though there is no evidence [effectiveness], but it is very difficult to disinvest, because our patients’ expectation is that, they need to be given some medication to treat their illness. (CLIN03)*

##### Perceived threats to authority

Concerns were also expressed about the perception of disinvestment as a criticism of decisions made by previous healthcare authorities. This view evokes discomfort or defensiveness, as it may suggest that previous decisions were misguided. Such interpretations foster a protective stance among stakeholders, who feel their professional autonomy and credibility are being challenged or questioned.

*…sometimes colleagues or staff may feel like resistance to change, particularly if reassessment or disinvestment perceived as criticism on any previous decision, or reduction in authority if someone were to give a decision on that. (CLIN04)*

Participants also feared negative public reactions, with potential blame directed at the MOH for perceived service reductions. This concern heightened reluctance toward disinvestment, as such decisions could expose the MOH to greater scrutiny and resistance.

#### Theme 3: Challenges and barriers to disinvestment

This theme highlighted key challenges and frustrations among healthcare professionals that hinder acceptance of disinvestment. Systemic issues such as inadequate planning, restrictions on investing in innovative technology, lack of funding, and bureaucratic challenges raise concerns about the system’s readiness and ability to manage disinvestment in a way that leads to significant improvement.

Stakeholders expressed doubts about the effectiveness of disinvestment in eliminating obsolete or LVC by replacing them with better alternatives or technologies. This uncertainty also contributes to apprehension and personal frustration among healthcare professionals, fostering hesitancy to shift from experience-based to evidence-based practices in the context of de-implementation. Table 2 presents the illustrative quotes associated with each subtheme, highlighting the barriers and challenges.

**Table 2.** Example of quotes on barriers to disinvestment initiatives

Subthemes	Example quotations
1. *Systemic barriers* *1.1. Poor planning and implementation*	*“One of the biggest hurdles from that process would be the implementation part. We do see very good policies, or a very good new method being introduced into the system. But along the way, implementation does not always turn out as good as expected. […] these transform into additional workload for the people who are involved.” (RSCH03)*
*1.2. Lack of funding to adopt high-value technology*	*“…they just assume that the government will give the budget that can cover 200 cases. I’m speaking about IVF only. […] If you look at the amount of budget received by each centre, there’s no way they can accommodate 200 cases!” (CLIN02)*
*1.3. Restriction to invest in innovative technology*	*“We can’t do a lot of things with that kind of restrictive policy. […] With that restrictive policy and the law, we feel that if we want to advance into new things [innovative technologies], sometimes are very limited.” (PRGM06)*
2. *Practical barriers* *2.1. Delay in technology adoption or change of practice*	*“When it comes to ART [artificial reproductive treatment] services, Malaysia is the pioneers in the Southeast Asia. But now we are being left behind by other countries, for example, Vietnam, Thailand, Singapore even more.” (CLIN02)*
*2.2. Outdated technology still being used*	*“It is sad for me now, because we see that even though we have been doing this for quite some time now, but it is very hard to say that we are growing. All of our colleagues that left the public sector, they manage to [grow] professionally, because they are able to use all the technologies that I mentioned.” (CLIN02)*
3. *Care provider’s apprehension* *3.1. Healthcare professionals’ frustration*	*“Some of my clinicians are frustrated, you know. If let’s say they stop something, and then they want to start something else. Like, they want to give new medication to the patients, they have to apply for permission… I think they are getting frustrated. It’s more of bureaucratic red tape that we need to get rid of, some of it.” (HOSP01)*
*3.2. Unsure of benefit* *3.3. Lack of knowledge/awareness*	*“They [healthcare professionals] will resist it, but it depends on how much they know about disinvestment. If* *we are in business operation, and we can say disinvestment is something that will benefit us, then it is good. But now, we do not really know how it will benefit us. If we do not know, how can we accept it?” (HOSP04)*
*3.4. Experience-based vs evidence-based* *3.5. Reluctant to change*	*“Sometimes for the very senior doctors, they rely on their experience. They have their own preference. For example, during COVID on the usage of steroids, they have different way [of managing patients] based on their experience*, *even we have guidelines [recommending oppositely]. They will not follow.” (HOSP04)*

#### Theme 4: Strategies for value assessment and effective implementation

This theme explores potential strategies to enhancing the acceptance and operationalization of disinvestment in the Malaysian healthcare system. Among the subthemes identified from the KII were suggestions to increase stakeholder acceptance through knowledge transfer, integrating change management strategies, including equity as one of the criteria for assessment, and looking into factors that can assist transitioning from low-value to high-value care.

##### Awareness, acceptance, and knowledge transfer

Educating stakeholders is crucial for successful disinvestment. Clear and transparent communication builds understanding and trust, and clinicians, as the key implementers, need clarity on the rationale to align their practice with disinvestment goals. A participant recounted prior experience engaging clinicians in the reassessment of drug lists and noted that:

*We have to tell them [doctors] that this kind of routine exercise [disinvestment] is actually for a greater good. It’s not for one perspective. We actually want to maximize what we have [resources and budget allocation]. (RSCH02)*

Other stakeholders that should be involved are patients. Building trust through open and transparent communication between doctors and patients can empower them to actively participate in their care and align their expectations with the broader goals of achieving value-based care. This approach offers an informed decision-making process for patients, thereby enhancing their acceptance of disinvestment.

*We have to educate them [patients] because they have the right to know why we decide on this. We have to respect their rights and their needs. (HOSP02)*

##### Change management strategies for disinvestment

In planning for disinvestment, robust change management strategy is vital because it involves withdrawing existing services, a process often more difficult than introducing new technologies. Stakeholders may perceive it as a loss rather than a strategic move toward value-based care. Change management strategy was specifically mentioned as a practical way to introduce disinvestment:

*Well, anything new is always difficult. You need your change management strategies to be in place. And you need to manage it in a stepwise manner as you go. Identify your champions, start with those champions, do with the smaller scope. Start with something very specific, and once you can show that it works or it can work well in one, then you start expanding it further. (PRGM01)*

Participants also emphasized that strong leadership is indispensable to driving disinvestment, ideally starting with a top-down approach or as a policy-driven effort. Framing the process as part of a quality improvement initiative can make it more acceptable to key stakeholders.

*Since this is a top-down approach versus a self-initiated bottom-up approach, there is a difference in terms of support. Once it’s a top-down approach, there’s more support. […] That is also the main reason that you can bring to your specialists and the consultants. (RSCH02)*

Participants underscored the significance of adaptable disinvestment implementation on a case-by-case basis, promoting localized decisions that consider facility readiness and the local context. Although a centralized policy on disinvestment might help in standardization, a blanket rule allowing all levels to implement disinvestment decision independently could lead to variability in care quality.

*It is very difficult to have a one-size-fits-all kind of decision, because a lot of decision we make […] should be customized to whatever is needed by the local population. (RSCH03)*

##### Equity criteria for assessment

Equity emerged as a key consideration in disinvestment decision making, with participants emphasizing the need to protect vulnerable populations and those with rare diseases. Some advocated for the inclusion of specific criteria to address emotional and sensitive issues, whereas others emphasized the importance of setting boundaries or safeguards to prevent disproportionate impact on these groups. However, they also acknowledged that disinvestment should not entirely exclude these underserved groups, but recommend a transparent, rationale-driven approach to assess interventions that may be ineffective or of limited benefit.

*We need to make sure that there are mitigating safeguards for the vulnerable population, by having exclusions and so on. Yes, there is a concern that it affects the vulnerable population. But as long as there are safeguards, then it should be implemented. (PRGM03)*

*We need to include assessment on equity as a criterion, because disinvestment is supposed to be transparent, and it’s supposed to be non-judgmental and also unbiased. If the evidence, the utilization and also the budget support that disinvestment [decision], I think we need to do that even though it involves the vulnerable groups. (CLIN03)*

##### Transitioning from low-value to high-value care

Transitioning from low-value to high-value care requires a comprehensive approach that considers healthcare system readiness, improvements in health financing mechanisms, and the acceptability of decisions for patients and the public. The suggestion to shift toward a more sustainable financing model was raised because the ability to effectively implement disinvestment is closely tied to how health systems pool and allocate resources. In a tax-funded system like Malaysia, limited fiscal space often constrains the reallocation of savings from disinvestment, whereas a more sustainable financing structure could strengthen strategic purchasing mechanisms, enabling disinvestment gains to be reinvested more transparently and systematically. It was suggested that although different countries may have different financing models, their functions can align through strategic purchasing, a financing function that operates similarly regardless of the broader funding model.

*We should be moving towards functions of our financing [system]. You were correct in saying that Vietnam has social health insurance and Malaysia has tax-funded. But when it comes to functions, both Vietnam and Malaysia […] can have in terms of functioning, strategic purchasing. The functions of purchasing are no different. (PRGM03)*

##### Monitoring and evaluation of implementation of health technologies

Monitoring and reassessment should be integral to health technology management to ensure care remains effective and relevant. Routine evaluation enables evidence-based updates and timely discontinuation of outdated practices, aligning healthcare delivery with evolving evidence and standards.

*If you make it a routine, it may be easier for colleagues or the staff to accept that whatever drugs come in, even the new drugs, they may be substituted for further reassessment along the way, although it has probably been included in this CPG. (PRGM04)*

## Discussion

This study employed qualitative methods to explore Malaysian stakeholder perspectives on the disinvestment of LVC. Four major themes were identified, including systemic and practical barriers, and apprehensions by care providers toward the process. The majority of the participants were keen on advancing this challenging process, taking into consideration the positive perceptions of how HTA is conducted in Malaysia, that is, using a systematic, transparent, and comprehensive approach involving key stakeholders. Some participants were skeptical, doubting whether the Malaysian healthcare system is ready in terms of the availability of skilled personnel and whether this process would jeopardize access to care and treatment options for underserved populations.

Despite diverse opinions, participants were unanimous in supporting equity as a key component in decision making for de-implementing LVC. This, however, contrasts with earlier online survey findings from 153 respondents that ranked equity or fairness as the least important element (10). We hypothesize that most healthcare stakeholders in the survey may find it challenging to grasp the rationale for prioritizing equity due to the complexity of disinvestment and its relative unfamiliarity.

### Stakeholders’ perceptions and engagement

Some stakeholders perceived disinvestment as a potential challenge to established authority, a view that reflects understandable uncertainties and misperceptions about its underlying purpose. Our study found that some perceived it as merely “unloading excess baggage” or cutting resources, rather than reallocating them to enhance value and efficiency. Several participants acknowledged that, in practice, not all disinvestment efforts are perceived as value-driven, which explains why some stakeholders suspect that disinvestment may occasionally be motivated by fiscal pressures rather than solely by improving efficiency or enhancing value.

Continuous awareness and education can gradually reshape stakeholders’ perceptions of disinvestment. A longitudinal study in two Australian hospitals found that staff initially associated disinvestment with a sense of loss but later viewed it as an opportunity to reallocate resources and improve care (14). This example demonstrates that showcasing clearly the benefits of disinvestment is vital to nurturing positive behavioral change and stakeholder support.

Clinicians often face pressure to provide certain treatments due to patient expectations (15), a challenge also noted by our participants. Removing established practices can threaten clinicians’ autonomy and be perceived as criticism of their professional judgment (16;17). Furthermore, due to the precedence of some legacy items and the lack of recent evaluation, LVC tends to persist through habit (18). Identifying inefficient care can be difficult, as some interventions may be effective for certain populations but not for others, leading to uncertainty. Hence, transparent and evidence-based identification using both top-down and bottom-up approaches is therefore essential.

### Change management strategy

A well-planned change management strategy is crucial for stakeholder acceptance and effective execution of disinvestment initiatives, as policies or guidelines alone are unlikely to succeed without strategic implementation planning (19). Applying change management models with sufficient flexibility to suit the specific context and empowering local change agents can enhance implementation outcomes (20). Moreover, strong leadership is fundamental to build trust, align stakeholders, and secure support from key public sector leaders whose influence can reduce resistance and foster acceptance of change (21).

Participants noted that poor implementation planning and inadequate funding often lead to stakeholder frustration and uncertainty about the benefits of disinvestment. An initial step could involve identifying champions among stakeholders committed to value-based practices. This can help advocate for the process, clarify why certain technologies are of low value, and ease concerns among implementers. Unlike introducing new services, disinvestment may entail a sense of loss or be perceived as a threat by those delivering care (14).

Policy makers are encouraged to enhance transparency and consistency in disinvestment decision making to build support and trust among stakeholders (22). Equally important is the need to monitor and evaluate the outcomes of disinvestment decisions to assess whether the program has achieved its intended objectives. These impacts can be measured using real-world data, such as claims records, updates to clinical guidelines, or surveys capturing public perspectives (23). Furthermore, our study highlighted the critical need for skilled personnel with proper training to carry out disinvestment assessments effectively. This involves not only improving their knowledge and technical competencies but also fostering collaboration with other stakeholders to address complex issues, particularly in ethical and social domains (24).

### Equity and protecting underserved population

One of the key insights from our study is the emphasis on preserving equity in disinvestment decision making. The majority of the participants agreed that safeguarding vulnerable populations is essential because people suffering from rare diseases, for instance, are already in a disadvantaged position at the starting point of their illness. Policy makers often assume they understand what these populations require, which may not align with their actual needs. Hence, including equity components and mitigation strategies in the decision-making process is vital to addressing their specific concerns and avoiding misrepresenting their priorities.

Our findings identified three key elements to ensuring equity for disadvantaged or vulnerable groups: *the availability of alternative services, accessibility across different levels of care and geographic regions, and the establishment of safeguards to protect these groups* *from inequitable outcomes.* These priorities highlight the importance of a framework that explicitly places equity at the core of disinvestment decisions. Failing to ensure the availability, accessibility, and acceptability of essential healthcare services for disadvantaged groups could compromise the quality of care, undermining the principles of universal health coverage (25).

### Strengths and limitations

Our study is the first to explore healthcare stakeholders’ perceptions of disinvestment in Malaysia through in-depth interviews. A key strength of this research is the diversity of participants, representing various professional roles across multiple levels of the healthcare system. Moreover, we included researchers with extensive experience in health services research, including conducting reassessments of medications listed in the national formulary. This is vital in identifying components for evidence generation and performing advanced technical analyses. Linking evidence on disinvestment with clinical practice recommendations can help ensure that practices remain contemporary and aligned with the latest evidence.

We acknowledge several limitations in this study. We conducted all interviews online, limiting our ability to observe non-verbal cues, especially when some participants chose not to use their cameras. We encountered technical issues during the interviews, including poor audio quality and internet connectivity. Despite these challenges, all interviews proceeded as planned. On the positive side, digital interviews offered significant advantages for both researchers and participants, enabling participation from their respective workplaces (26) and facilitating data collection across geographic boundaries. As in previous studies, Zoom emerged as a practical tool for qualitative data collection due to its user-friendly interface; cost-effective, robust data management features; and reliable security options (27;28).

Although recruiting participants through purposive sampling ensured the inclusion of experienced and knowledgeable participants, it may have excluded perspectives from other healthcare providers who may hold different views or face particular challenges. Additionally, the use of snowball sampling, which relies on recommendations from professional networks, likely favored respondents with extensive experience and familiarity with the healthcare system. Consequently, some degree of selection bias cannot be entirely excluded.

## Conclusion

In this study, Malaysian healthcare stakeholders viewed disinvestment both optimistically as a catalyst for efficient resource allocation and skeptically as a possible justification for budget reductions in health care. Key challenges identified included apprehension about its potential negative consequences and concerns regarding the Malaysian healthcare system’s readiness to implement disinvestment effectively. We identified potential strategies for value-based assessment and implementation to address these barriers, emphasizing the importance of fostering accountability and collaboration among stakeholders. To date, disinvestment efforts in Malaysia have been largely limited to the pharmaceutical domain, mainly through rationalization of drug lists for similar indications, though such initiatives remain undocumented. The findings from this study may therefore provide transferable insights for other countries and healthcare systems seeking to formalize structured, transparent, and equitable processes for disinvesting from LVC. The thematic framework developed in this study, which captures stakeholders’ perspectives on resource allocation, budget pressures, implementation challenges, and strategies for effective change, offers a structured lens for assessing contextual readiness. These stakeholder-derived themes and subthemes may serve as a practical guide for healthcare settings aiming to design context-sensitive, equitable, and sustainable approaches to disinvestment.

## Supporting information

10.1017/S0266462325103437.sm001 Kamaruzaman et al. supplementary material Kamaruzaman et al. supplementary material

## Supplementary material

The supplementary material for this article can be found at http://doi.org/10.1017/S0266462325103437.
