# Supplementary material for: Unloading the excess baggage: key informant interviews with Malaysian stakeholders on healthcare disinvestment initiatives
Source: Int J Technol Assess Health Care. 2026 Jan 5;42(1):e6. doi: 10.1017/S0266462325103437 (PMC12835830; doi:10.1017/S0266462325103437)
Supplement: Kamaruzaman et al. supplementary material [file S0266462325103437sup001.pdf]

SUPPLEMENTARY DOCUMENTS

Unloading the excess baggage: Key informant interviews with Malaysian stakeholders on healthcare disinvestment initiatives

Supplement 1: Key informant interview guide

Part A: Introduction

- *(Greeting and thank the participant for agreeing to involve in the interview)*
- As was described in the information sheet, this research concerns disinvestment which involve any existing healthcare practices, procedures, technologies, or pharmaceuticals. In this study, disinvestment includes:
  i. **removal** or withdrawal of resources, either full or partial disinvestment
  ii. the **restriction** of reimbursement or funding, such as for certain group of patients with specific criteria
  iii. the **retraction** of budget or funding from **reimbursement**
  iv. the **replacement** of healthcare interventions by alternatives

- To be able to analyse the interview later, I will record this using Zoom video recording. However, only the audio recording will be retained for further process, transcribing and analysis.
- I will send you the transcribed interview, so that you can check for any inaccuracy or misinformation. The research team will handle the audio recordings confidentially. Only the research team will have access to these files.
- The results of this study may be published and presented in scientific meetings or conferences. We may use any of your quotes in these publication or presentations to substantiate the results. We will, however, ensure that both the results and the quotes will not contain information that may reveal your identity.
- Do you agree with:
  - Participation in this study?
  - The video / audio recording and writing down of the interview?
  - The inclusion of the results and possible quotes in a publication?

- Can you please tell me briefly on your background information, experience and your roles in healthcare system?

Part B: Interview Questions

1. **Assessment Method**
   a. Are you familiar with any method or process for priority setting and resource allocation within your organisation / level of governance you are involved in?
   b. What do you think are the criteria involved for disinvestment or decision to stop funding any practices or use of technologies / drugs? Is there a formal process or framework in place for this?
   c. What do you think is the underlying rationale or principles in disinvestment that must be prioritise? (*even if a formal process does not exist yet*)

2. **Evidence & Data**
   a. In the absence of robust data (eg. published evidence or data collected for clinical trials), would you be confident to use RWE for disinvestment purposes?
   b. Apart from this, what do you think constitutes optimal evidence for reassessment of obsolete technologies or disinvestment of low-value care?
   c. In your opinion, should Axitinib and Everolimus be included in the formulary? If yes, what are the method that can be used to assess for listing in the formulary / new indications for clinical practice?

3. **Stakeholder Involvement**
   a. In our survey, the majority agreed that reassessment or disinvestment process should be done routinely as part of monitoring the decisions that was done before. What do you think are the impacts of implementing this process in terms of acceptance from your staff / colleagues and the benefits of it?
   b. Do you think public / patients should be involved actively in this process? At which level and how?

4. **Equity / boundary in disinvestment**
   a. Should we set a 'boundary' or exclusion to implement disinvestment process in the areas that are considered as **emotive / sensitive**, such as end-of-life care, **rare diseases** and **vulnerable populations** (children, terminally ill, elderly?)

5. Implementation

a. Should the disinvestment decisions that have been implemented / approved in other countries be adopted to the Malaysian context in terms of service provision?

b. What is / are your biggest concern when it comes to the implementation of a potential disinvestment decision?

Part C: Closing

- Are there other things you would like to discuss about disinvestment processes/ initiative? Are there any relevant issues that have not yet been discussed?
- Are there other stakeholders you think I should interview about this? If so, who could we approach?
- *(If no more questions, thank the respondent and close session)*

Supplement 2: Consolidated criteria for reporting qualitative studies (COREQ): 32-item checklist

Developed from:
Tong A, Sainsbury P, Craig J. Consolidated criteria for reporting qualitative research (COREQ): a 32-item checklist for interviews and focus groups. International Journal for Quality in Health Care. 2007. Volume 19, Number 6: pp. 349 – 357.

Item No	Guide Questions/Description	Reported Page #
**Domain 1: Research team and reflexivity**		
**Personal Characteristics**		
1. Interviewer/ facilitator	Which author/s conducted the interview or focus group?	2
2. Credentials	What were the researcher's credentials? E.g., PhD, MD	2
3. Occupation	What was their occupation at the time of the study?	2
4. Gender	Was the researcher male or female?	2
5. Experience and training	What experience or training did the researcher have?	2
**Relationship with participants**		
6. Relationship established	Was a relationship established prior to study commencement?	N/A
7. Participant knowledge of the interviewer	What did the participants know about the researcher? e.g. personal goals, reasons for doing the research?	2
8. Interviewer characteristics	What characteristics were reported about the interviewer/facilitator? e.g. Bias, assumptions, reasons and interests in the research topic	2
**Domain 2: study design**		
**Theoretical framework**		
9. Methodological orientation and Theory	What methodological orientation was stated to underpin the study? e.g. grounded theory, discourse analysis, ethnography, phenomenology, content analysis	2
**Participant selection**		
10. Sampling	How were participants selected? e.g., purposive, convenience, consecutive, snowball	2
11. Method of approach	How were participants approached? e.g., face-to-face, telephone, mail, email	2
12. Sample size	How many participants were in the study?	2 & 3

Item No	Guide Questions/Description	Reported Page #
13. Non-participation Setting	How many people refused to participate or dropped out? Reasons?	12
14. Setting of data collection	Where was the data collected? e.g., home, clinic, workplace	10 & 11
15. Presence of nonparticipants	Was anyone else present besides the participants and researchers?	10
16. Description of sample	What are the important characteristics of the sample? e.g. demographic data, date	9 & 10
**Data collection**		
17. Interview guide	Were questions, prompts, guides provided by the authors? Was it pilot tested?	3, Suppl. 1
18. Repeat interviews	Were repeat interviews carried out? If yes, how many?	N/A
19. Audio/visual recording	Did the research use audio or visual recording to collect the data?	2
20. Field notes	Were field notes made during and/or after the interview or focus group?	N/A
21. Duration	What was the duration of the interviews or focus group?	2
22. Data saturation	Was data saturation discussed?	N/A
23. Transcripts returned	Were transcripts returned to participants for comment and/or correction?	2
**Domain 3: analysis and findings**		
**Data analysis**		
24. Number of data coders	How many data coders coded the data?	2
25. Description of the coding tree	Did the authors provide a description of the coding tree?	3 & 4
26. Derivation of themes	Were themes identified in advance or derived from the data?	2 & 3
27. Software	What software, if applicable, was used to manage the data?	2
28. Participant checking	Did participants provide feedback on the findings?	N/A
**Reporting**		
29. Quotations presented	Were participant quotations presented to illustrate the themes/findings? Was each quotation identified? e.g., participant number	3 - 7
30. Data and findings consistent	Was there consistency between the data presented and the findings?	3 - 7
31. Clarity of major themes	Were major themes clearly presented in the findings?	3 - 7
32. Clarity of minor themes	Is there a description of diverse cases or a discussion of minor themes?	N/A
